# Portuguese Primary Healthcare and Prevention Quality Indicators for Diabetes Mellitus – A Data Envelopment Analysis

**DOI:** 10.34172/ijhpm.2021.76

**Published:** 2021-07-26

**Authors:** Andre Ramalho, Julio Souza, Pedro Castro, Mariana Lobo, Paulo Santos, Alberto Freitas

**Affiliations:** ^1^MEDCIDS - Department of Community Medicine, Information and Health Decision Sciences, Faculty of Medicine, University of Porto, Porto, Portugal.; ^2^CINTESIS - Centre for Health Technology and Services Research, Porto, Portugal.; ^3^USF Camélias, ACES Gaia (Grande Porto VII - ARS Norte), Vila Nova de Gaia, Portugal.

**Keywords:** Diabetes Mellitus, Health Policy, Primary Care, Preventable Admission, Efficiency, Data Envelopment Analysis

## Abstract

**Background:** Diabetes mellitus (DM) is a worldwide public health priority. The increasing prevalence and the budget constraints force to have effective healthcare, especially at the primary healthcare (PHC) level. We aim to assess primary care efficiency considering the best use of human resources to produce optimal diabetes care in terms of prevention quality indicators (PQIs) rates across national ACES (health centre groupings).

**Methods:** We conducted a two-stage data envelopment analysis (DEA) to assess the technical efficiency of 54 Portuguese primary care health centre groupings for the 2016-2017 biennium. In the first stage, efficiency scores were obtained through five output-oriented DEA models under vector return to scale (VRS) assumption, using three input variables representing key primary care human resources and one output representing each one of the five PQIs related to diabetes. In the second stage, Tobit regression models were estimated to assess the determinants of primary care efficiency in diabetes care.

**Results:** A total of 13 ACES reached the efficiency frontier. Better managing human resources could reduce PQI rates by 52.3% in 2016 and 49.1% in 2017. Higher proportion of patients under 65 years old and better controlled with a hemoglobin A1c (HbA1c) ≤6.5% were associated with better efficiency in diabetes care, whereas higher prevalence of DM and unemployment worsened hospitalizations rates by diabetes short-term complications and lower-extremity amputation.

**Conclusion:** Inefficiency in DM care was found in most of the primary care settings which can substantially improve the avoidable hospitalization rates by DM using their current level human resources. These findings help to improve diabetes care by targeting human resources at primary care level, which should be integrated into performance assessments considering broader and integrated scopes.

## Background

**Key Messages**

** Implications for policy makers**

- Data envelopment analysis (DEA) makes it possible to objectively compare the measured results and can therefore be considered by countries and organizations, including local governments responsible for implementing and improving sustainable development policies.
- It is a useful tool for facilitating an objective assessment of the effects resulting from treatment and control actions directed at diabetic patients.
- Efficiency assessment is a useful way to obtain insights into providers’ needs and potential of improvement in diabetes care at primary care level, and thus could be integrated into healthcare delivery performance assessments considering broader and integrated scopes.

** Implications for the public**

Health quality indicators are a set of essential tools for monitoring public policies not only at the level of policymakers, but also in signalling the effective control of diabetes mellitus (DM), a chronic condition of important prevalence nowadays. The study of efficiency evidenced in this study makes the performance of primary healthcare (PHC) compared to the outcomes presented by hospital indicators of preventable hospitalizations. The identification of the determinants for diabetes that influence greater variability in the negative outcome evaluated allows us insights for better management and monitoring of patients in order to avoid complications and hospitalizations, promoting better quality of life and consequently reducing related costs.

Over the last decades, we have seen a great effort by governments and health authorities to improve overall quality of health services, especially in primary healthcare (PHC), the pillar of the entire health system.^1-3^ This is a relevant concern for policy-makers as better primary care services, which comprises health promotion, prevention and monitoring of health-disease processes and the natural history of co-morbidities, have an impact on overall populations’ health, whether they are favourable or not.^4-6^ A study was carried out previously by the authors that synthesised and categorised the quality indicators frequently used and monitored by PHC, published in peer-reviewed studies indexed in bibliographic databases.^7^ Quality indicators are a set of measures that are especially useful in monitoring processes and results, controlling compliance with best clinical practices through quantitative parameters.^8,9^ Thus, this monitoring through indicators aims at better health processes and outcomes. The growing number of patients suffering from chronic diseases,^10,11^ mostly as a result of aging populations, have forced healthcare systems to address increasing costs and demand for care. An important share of healthcare spending is considered ineffective and wasteful, mostly due to ambulatory care sensitive conditions or avoidable hospitalizations. A large number of hospital admissions could be avoided through better prevention and management of chronic conditions at primary care level. Amongst more than 30 conditions for which hospitalization could be reduced with improved primary care,^12^ diabetes mellitus (DM) stand out as particularly relevant in European countries, being responsible for more than 800 000 admissions in the European Union in 2015, consuming more than 6.7 million bed days (1.1% of all bed days).^13^

 DM is a chronic disease where the need of higher quality of primary care is paradigmatic,^14^ due to the worldwide high prevalence, the inherent complexity that implies greater proximity to the patient, and the possibility to prevent most of the complications causing hospitalization.^15-18^

 In Portugal, 9.2% of the population lived with DM in 2016, which caused 5% of all deaths.^16,19,20^ Therefore, tracking preventable hospitalizations due to diabetes is a key strategy to indirectly evaluate primary care performance regarding this disease. The Agency for Healthcare Research and Quality developed the prevention quality indicators (PQIs), which consist in a set of measures that can be used with hospital discharge data to identify the performance of primary care regarding the treatment and management of ambulatory care sensitive conditions.^7,21-25^ Despite recent improvements, preventable hospitalizations rates due to diabetes in Portugal remain high and vary substantially across local and regional providers, hence monitoring is of utmost importance. The total number of hospital admissions due to diabetes complications was 8.5 admissions in 2016, decreasing to 6.9 admissions in 2017, with long term diabetes complications being the main contributor to these results (4012 hospitalizations in 2016 and 2639 hospitalizations in 2017).^26^ However, during that period, hospitalizations due to uncontrolled diabetes and lower-extremity amputation among patients with diabetes complications increased, indicating that some areas of diabetes care need to be improved. As the performance of PHC reflects onto hospitalizations for conditions sensitive to this level of care,^27-29^ thus efficiency in primary care can reduce this type of hospital admissions, thereby reducing wasteful spending on healthcare.

 In 1999 the Institute of Medicine defined quality of care as “the degree to which health services increase the likelihood of desired results and are consistent with current professional knowledge,”^30^ and categorised the domains of health quality in 2001 into six pillars: safe, effective, efficient, timely, patient-centred, and equitable.^31,32^ We note, however, that efficiency assessment specially in PHC is still an under-explored pillar.

 Over the last decades, several techniques to perform efficiency analysis in healthcare have been proposed,^33^ with a predominant use of data envelopment analysis (DEA) in nearly all healthcare sectors.^34^ Regarding primary care, DEA has increasingly been employed, showing its suitability for this setting.^35^ DEA is a non-parametric technique that was first presented in the study “Measuring the efficiency of decision-making units (DMU)” in 1978.^36^ Several studies were subsequently carried out, mainly in the areas of management, engineering and healthcare, the last one often with few consensuses regarding the most appropriate method.^37-44^ Nevertheless, authors prefer the use of DEA methods due to many underlying advantages, namely the possibility of considering multiple inputs and outputs, which better characterize the health production process, the fact that it does not require a mathematical specification or assumptions regarding the production function, apart from being the most appropriate approach to assess the impact of exogenous variables and for providing means to draw recommendation and potential of improvement for inefficient units.^45^ Healthcare efficiency has been increasingly assessed over time and more recently has been considered a relevant quality indicator for healthcare assessment, including PHC.^7,46^ Some studies were carried out involving the analysis of efficiency through DEA, specifically in the context of DM, but did not evaluate the unfavourable outcome (preventable hospitalizations) as an output.^43,47-51^ Since efficiency can be considered a polysemantic word, in this study we considered the definition of efficiency as “the capacity or even the ability to make the most appropriate use of the resources available to achieve an intended result.”^52,53^ To fill this gap, our study aims to evaluate the efficiency of primary care in Portugal regarding diabetes care, considering the broadest perspective of policy-makers and public health managers, that is, how to use the available resources to reduce avoidable hospitalizations due to DM, which represents a proxy of overall PHC quality concerning this particular disease.

## Methods

###  Data Sources and Variables

 The Portuguese PHC is backed up by a robust information system, producing a comprehensive set of primary care activity and performance indicators that are made publicly available by the Ministry of Health at the *Bilhete de Identidade dos Cuidados de Saúde Primários* website, and the definitions of these indicators can be found elsewhere.^20,55^ Data on human resources to be used as input variables to the DEA models, as well as variables for Tobit analysis were extracted from these sources. In mainland Portugal, the primary care is organized into five regions called Health Regional Administration, which are responsible for tackling the health needs of the population living in their area of intervention, including resource management and actions to ensure the access and quality of health services. Each health regional administration is composed of several primary care health centre groupings denominated ACES (*Agrupamentos de Centros de Saúde*), which in turn provide care to the population living within a specific geographical area of intervention, usually at municipality level.

 To estimate diabetes-related PQI rates, we used patient level data abstracted from the Portuguese National Hospital Morbidity Database, which contains data on inpatient and outpatient episodes that occurred in all public hospitals within the Portuguese National Health Service. Coded clinical data from all inpatient episodes with a discharge date between January 1, 2016 and December 31, 2017 were extracted. Variables included diagnoses and procedures coded according to the International Classification of Diseases, Clinical Modification, ninth and tenth revisions (ICD-9-CM/ICD-10-CM); age; sex; discharge status and place of residence. The latter variable was included to assign each inpatient episode to an ACES. We identified avoidable hospitalizations according to the definitions of all 5 DM-related PQIs reported in the version 6.0 for ICD-9-CM codes and version 2019 for ICD-10-CM/PCS codes, both available at the US Agency for Healthcare Quality documentation.^23^ Age-sex standardized PQI rates were calculated for each ACES using the direct standardization method, considering population by age and sex at parish level collected from the 2011 Census, which is the latest population census available by the Portugal’s National Institute of Statistics.^56^

 The variables analyzed in this study include Input variables (number of doctors, number of nurses and number of intern doctors), Output variables (PQI 01: Diabetes Short-Term Complications Admission Rate, PQI 03: Diabetes Long-Term Complications Admission Rate, PQI 14: Uncontrolled Diabetes Admission Rate, PQI 16: Lower-Extremity Amputation Among Patients with Diabetes Rate, and PQI 93: Prevention Quality Diabetes Composite), and the Environmental variables (Ratio Patients by Medical Doctors, Proportion of elderly patients, Unemployment rate, Prevalence of DM, Proportion of patients with DM and record of hemoglobin A1c (HbA1c) in the last six months, Proportion of patients with DM and with last HbA1c ≤8.0%, Proportion of patients with DM <65 years old with a HbA1c test ≤6.5%, Proportion of patients with DM and with a microalbuminuria test in the last year, Proportion of patients with a risk assessment for DM2 (3 years), Cost of therapy for diabetic patients, and Cost of therapy for diabetic patients under control). Before describing the employed chosen model, it is worth mentioning that efficiency can be assessed in health outcomes and health outputs.^31,33^ Specifically, our work assesses the efficiency based on health outcomes in primary care, which are usually represented by preventable admissions and which, as already explained, are usually considered as a quality indicator of PHC. Efficiency was analysed, particularly in the context of primary care related to diabetes care.

###  Data Envelopment Analysis Model

 In DEA efficiency assessments, the units of analysis are denominated DMU, which in our study are represented by the 55 ACES in mainland Portugal. The relative efficiency of ACES was evaluated by means of multiple inputs (PHC resources) and multiple products (PHC outcomes). The objective of our DEA model, therefore, is to compare the 55 ACES that perform similar tasks (PHC) and differ in the quantities of inputs (human resources) they provide and the outputs they produce (diabetes PQIs results). Nevertheless, ACES Pinhal Interior Sul was excluded from analysis as it operates under different conditions in comparison with the other ACES, namely due to the smaller size of the population covered, and for being the only ACES that do not present intern doctors in its human resource composition. Therefore, the final sample comprises 54 DMUs.

 Relevant points to choose the DEA model, among others, are the possibility of the model to deal with the existence of several non-proportional inputs and outputs; to use all available data in order to build an empirical frontier of best practices, in which each production point classified as “not optimal” is compared; it does not require the specification of the functional form that connects inputs to outputs; and the performance of the units can be assessed through several alternative guidelines for the frontier of best practices, depending on the context of the study. Despite these advantages, the model can also bring some disadvantages. These were considered when conducting this study and mitigated by the close engagement with the practices. Disadvantages include the fact that no DEA model can include all the potential variables (can lead to partial and potentially misleading results), and standard DEA models do not consider stochastic variability in the data (susceptible to data errors).^39,47,57^

 There are two approaches to DEA models, the first approach is classically known as VRS (vector return to scale, or Banker, Charnes and Cooper), which considers variable returns to scale and does not assume proportionality between inputs and outputs; and the second approach, the CRS (constant return to scale, or Charnes, Cooper and Rhodes scale -), which considers constant scale returns and that any variation in inputs leads to a proportional variation in products.^36^ As we aim to assess efficiency under management perspective, where the extent to which the scale of operations or different practices and primary care providers’ styles affect efficiency in PHC delivery, we opted for the VRS assumption.^33,35^

 The other approach to be considering in DEA refers to the efficiency analysis orientation, which typically can be input-oriented or output-oriented. In input-oriented DEA, the linear programming model determines how much the input use of a DMU could contract when used efficiently to achieve the same levels of output. In this sense, the input-oriented DEA is less relevant for estimating capacity utilization. On the other hand, in the output-oriented DEA, the linear programming model determines the DMU’s potential output given its levels of inputs, when operating efficiently (along with the best practice frontier). In other words, the output-oriented approach estimates the potential output for a given level of inputs and can be used to measure capacity utilization by means of the ratio between the actual to potential outputs. In the context of this study, the definition of the efficiency analysis orientation should be based upon what primary care managers are able to control better, either their already limited resources or health outcomes.^52^

 In fact, under a moral perspective, healthcare facilities should not aim to reduce inputs and costs but promote priority-setting and thus concentrate resources on improving health outcomes (outputs). Therefore, output-oriented is the model that best fits the perspective of this study, in which an ACES is made efficient through the proportional increase of the outputs while keeping its inputs unchanged. Nevertheless, as the output considered are avoidable diabetes hospitalization rates, the models included the inverse of the rates as outputs, in line with an approach conducted by Sahin and Ozcan.^53^ This procedure was applied because the objective of the ACES should be to decrease the negative outcome (preventable hospitalizations) and not to reduce the human resources allocated at this level of care, given the scarcity of resources and the shortage of professionals in the health sector, as discussed by several authors.^58,59^ Thus, it is intended that, with the same level of human resources in each ACES, better health indicators could be produced for the population (lower rates of preventable hospitalizations due to diabetes complications).

 The input variables chosen to represent the models reflect human resources present in each ACES to provide care to their respective area of intervention. These input variables were represented by the number of medical doctors, number of resident doctors and number of nurses. The output variables to be included were the inverse of five PQI related to preventable diabetes hospitalizations^23^: PQI 01 - Diabetes Short-Term Complications Admission Rate; PQI 03 - Diabetes Long-Term Complications Admission Rate; PQI 14 - Uncontrolled Diabetes Admission Rate; PQI 16 - Lower-Extremity Amputation Among Patients with Diabetes Rate: and PQI 93 - Prevention Quality Diabetes Composite. We considered separated models to assess each individual PQI and the composite indicator as each PQI reflects a distinct condition related to diabetes, despite possible correlations between them. Such conditions may differ in terms of prevalence and severity. In this sense, a primary care provider that performs well for a given condition may not perform equally well for another. Thereby, evaluating PHC efficiency for individual PQIs was the most appropriate approach to provide results that are clinically relevant and to identify which diabetes-related complications or conditions need to be addressed more urgently at primary care level.

 Finally, as we considered the inverse of the PQI rates as output to the DEA models, some unbalanced scale issues affecting DEA estimates may arise as the resulting outputs assumed values that were lower than 1, contrasting with the input variables, which are counts of healthcare professionals. Therefore, we performed mean normalization to address this problem, as suggested in appropriate literature,^60^ which can be described with the following general equation:


$$vNORM_{ai}=\frac{v_{ai}}{{\bar{v}}_{i}}$$

where, *vNORM*_ai_ is the normalized value ${\bar{v}}_{i}$ of the input or output variable *i*associated with DMU *a,* is the mean value of input or output *i* for the 54 ACES and *v*_ai_ represents the observed value of the input or output *i* for ACES *a.*

###  Tobit Regression

 Tobit regression was performed using the efficiency scores obtained with the application of DEA as dependent variable to investigate the several factors influencing inefficiency in PHC. The tobit model is adopted when the dependent variable is observed only within a specific numerical range caused by a form of censorship in the observations. In this case, the estimation by ordinary least squares (OLS) does result in inconsistent estimates of the parameters, since the restricted range of the dependent variable can make the censored sample not representative of the population.^61^ Moreover, efficiency scores obtained with DEA consist in a relative rather than an absolute index, and thereby are correlated between them, making the OLS regression invalid.^62^ Therefore, Tobit regression, which is one of few dependent variable models addressing a censored structure,^34^ is performed in this study using the maximum likelihood estimation method for parameter estimation.

 It intended to discover which factors explain a larger proportion of the variability of the efficiency scores controlling for external factors in each ACES. The factors that could potentially influence efficiency in diabetes care were: Ratio of patients by medical doctors, Prevalence of DM, Proportion of elderly patients and unemployment rate, Proportion of patients with DM and record of HbA1c in the last six months, Proportion of patients with DM and with last HbA1c ≤8.0%, Proportion of patients with DM <65 years old with a HbA1c test ≤6.5%, Proportion of patients with DM and with microalbuminuria test in the last year, Proportion of patients with a risk assessment for diabetes type 2 (3 years), Adequate follow-up index of patients with DM, Cost of therapy for diabetic patients, and Cost of therapy for diabetic patients under control.

## Results

###  Data Envelopment Analysis Model

 A total of five DEA models were estimated for cross-sectional data of both years (2016 and 2017), each of which considering the five distinct PQIs related to diabetes as outputs. Furthermore, Tobit regression was applied in the second stage to identify the determinants (external or environmental factors) affecting the efficiency scores obtained in the first stage. Table 1 presents the descriptive statistics for inputs, outputs, and environmental variables of the 54 Portuguese health centre groupings (ACES) included in our sample.

**Table 1:** Descriptive Statistics of the Input, Output and Environmental Variables at ACES Levels (years 2016 and 2017)

	**Mean (SD)**	
	**2016**	**2017**
Input variables		
Number of doctors	101.1 (42.8)	104.5 (44.1)
Number of nurses	110.2 (41.4)	111.1 (41.7)
Number of intern doctors	33.5 (19.3)	36.1 (20.1)
Output variables		
PQI 01: Diabetes short-term complications admission rate	19.5 (8.7)	14.6 (5.9)
PQI 03: Diabetes long-term complications admission rate	40.0 (14.5)	27.1 (11.0)
PQI 14: Uncontrolled diabetes admission rate	12.6 (8.7)	15.4 (9.8)
PQI 16: Lower-extremity amputation among patients with diabetes rate	13.1 (5.7)	11.2 (5.2)
PQI 93: Prevention quality diabetes composite	82.8 (26.2)	66.2 (20.2)
Environmental variables		
Ratio patients by medical doctors	1684.4 (119.6)	1660.4 (167.7)
Proportion of elderly patients	22.5 (4.0)	22.7 (3.9)
Unemployment rate	7.4 (1.6)	7.4 (1.6)
Prevalence of DM	8.2 (1.1)	8.3 (1.1)
Proportion of patients with DM and record of HbA1c in the last 6 months	67.1 (12.1)	66.9 (11.4)
Proportion of patients with DM and with last HbA1c ≤8.0%	58.8 (11.2)	58.4 (10.5)
Proportion of patients with DM <65 years old and HbA1c ≤6.5%,	27.7 (7.5)	27.5 (7.2)
Proportion of patients with DM and with microalbuminuria test in the last year	62.5 (14.8)	62.5 (14.1)
Proportion of patients with a risk assessment for DM2 (3 years)	18.4 (12.2)	24.6 (11.9)
Cost of therapy for diabetic patients (€/patient)	287.5 (41.1)	323.0 (39.8)
Cost of therapy for diabetic patients under control (€/patient)	291.4 (45.6)	325.3 (42.6)

Abbreviations: SD, standard deviation; ACES, Health Center Groupings; PQI, prevention quality indicator; DM, diabetes mellitus; HbA1c, hemoglobin A1c.

 In the first stage, basic radial DEA models were run to estimate efficiency, which consists of computing the ratio between outputs and inputs and comparing it with a benchmark. Fully efficient ACES are those with an efficiency score equal to 1, whereas values above 1 represent inefficiencies in the utilization of human resources to produce low PQI rates. Table 2 shows the mean efficiency scores for each PQI and year, considering the five output-oriented DEA models (one for each PQI) under VRS assumption. A total of 13 ACES reached the efficiency frontier in at least one PQI in a given year (Supplementary file 1, Table S1). The rate of ACES with efficiency scores above the MES in 2016 was 40.7, 46.3, 38.9, 46.3 and 51.9% for PQIs 01, 03, 14, 16 and 93, respectively. In 2017, this rate was found to be 50.0, 51.9, 44.4, 40.7 and 46.3% for PQIs 01, 03, 14, 16 and 93, respectively. The individual efficiency scores per ACES can be found in Supplementary file 1.

**Table 2:** Estimation of the National Overall MES Using VRS DEA Model With Output Orientation for 2016 and 2017

**PQI**	**2016 (MES)**	**2017 (MES)**
PQI 01: Diabetes short-term complications admission rate	2.91	3.13
PQI 03: Diabetes long-term complications admission rate	2.11	3.55
PQI 14: Uncontrolled diabetes admission rate	10.47	6.65
PQI 16: Lower-extremity amputation among patients with diabetes rate	2.35	3.29
PQI 93: Prevention quality diabetes composite	1.99	1.92

Abbreviations: MES, mean efficiency score; DEA, data envelopment analysis; VRS, vector return to scale; PQI, prevention quality indicator.

 Considering the VRS assumption, an investigation into the input slacks for PQI 93 in 2016 revealed slacks in the number of doctor and nurses for 49 ACES, whereas input slacks in the number of intern doctors were identified for 44 ACES in 2016. In the following year, input slacks for doctors, nurses and intern doctors were found for 48, 35 and 40 ACES, respectively. Table 3 shows the input slacks averages for each year when using PQI 93 as output. The highest slack values were found for doctors and nurses, even though a considerable improvement was observed in 2017 for all professionals. Overall, these results indicate that most of the ACES have a substantial slack capacity in their health workforce.

**Table 3:** Mean Input Slacks of the ACES, Model PQI 93 (DM Prevention Composite Indicator)

	**VRS DEA Model**	
	**2016**	**2017**
Doctors	63.33	32.02
Nurses	71.55	28.67
Interns	20.13	16.73

Abbreviations: DEA, data envelopment analysis; VRS, vector return to scale; PQI, prevention quality indicator; ACES, Health Center Groupings; DM, diabetes mellitus.

 Assuming the efficiency scores obtained with each DEA model as dependent variables, tobit regression censored at 1 was performed to assess the influence of environmental determinants on PHC efficiency in diabetes care. The estimation results of the Tobit fixed effects are presented in Table 4. The interpretation of the coefficients is similar to the OLS regression ie, how much the efficiency score can be increased or reduced following the one-unit increase of the independent variable. Residual analysis suggests good fit the models (Supplementary file 1). Five models were estimated considering the efficiency scores obtained for each PQI in 2017. Environmental variables for 2016 were included in the models as environmental determinants, as a lag of one year was assumed to examine the effects of such determinants on efficiency in diabetes care. A positive and statistically significant relation between efficiency and proportion of patients with DM <65 years old with a HbA1c test ≤6.5% was found for PQIs 03, 16 and 93, indicating that such control is important for improving efficiency scores (positive relation indicates decrease in the efficiency scores). On the other hand, a negative and statistically significant relation was found between efficiency and prevalence of DM and unemployment rate for PQI 1 and PQI 16, respectively. Moreover, a positive and statistically significant relation with efficiency was also found for proportion of elderly patients for PQIs 01.

**Table 4:** Analysis of PHC Efficiency Determinants in DM Care According to Tobit Regressions Estimates

**Variables**	**PQI 01**	**PQI 03**	**PQI 14**	**PQI 16**	**PQI 93**
Ratio patients by medical doctors	-0.00273	-0.00449	0.00020	-0.00085	-0.00047
Proportion of elderly patients	-0.22080^a^	-0.26968	-0.27967	-0.17928	-0.07842
Unemployment rate	0.00621	-0.15320	-0.79311	0.06988^a^	0.00204
Prevalence of DM	0.37458^a^	0.46684	0.85970	0.04491	0.21889
Proportion of patients with DM and record of HbA1c in the last 6 months	-0.34801	-0.07783	-0.74889	-0.33711	-0.14152
Proportion of patients with DM and with last HbA1c ≤8.0%	0.47534	0.14058	0.92115	0.37986	0.16523
Proportion of patients with DM <65 years old and HbA1c ≤6.5%,	-0.33089	-0.22754^a^	-0.43825	-0.31056^a^	-0.10829^b^
Proportion of patients with DM and with microalbuminuria test in the last year	0.01837	0.03322	0.16102	0.07323	0.01817
Proportion of patients with a risk assessment for DM2 (3 years)	0.01041	-0.04713	-0.08536	-0.03097	-0.00877
Cost of therapy for diabetic patients (€/patient)	0.02168	0.02334	-0.04619	0.03490	0.00860
Cost of therapy for diabetic patients under control (€/patient)	-0.02127	-0.02407	0.03495	-0.03029	-0.01007

Abbreviations: PHC, primary healthcare; PQI, prevention quality indicator; DM, diabetes mellitus; HbA1c, hemoglobin A1c. ^a^ *P* <.05; ^b^*P* <.01.

 When assessing the potential of improvements considering the fully efficient ACES as references for allocating human resources, a substantial improvement (reduction) in standardized PQI rates can be verified according to the models. These values represent optimal standardized PQI rates in a scenario in which inefficient ACES are placed in the efficiency frontier considering the combination of input and output values of the fully efficient ACES. Table 5 indicates, for each PQI, the target values for the output variable, represented by the optimal values of standardized PQI rates in 2016 and 2017. Regarding the composite indicator (PQI 93), the model estimated a reduction from an average of 82.8 to 43.3 hospitalizations per 100 000 inhabitants (reduction of 47.3%) in 2016, and from 66.2 to 35.6 hospitalizations per 100 000 inhabitants (reduction of 46.2%) in 2017. The remaining specific PQIs related to diabetes could also be reduced by a wide margin in both 2016 (PQI 01: -62.6%; PQI 03: -49.3%; PQI 14: -81.0%; PQI 16: -55.0%) and 2017 (PQI 01: -65.1%; PQI 03: -66.4%; PQI 14: -82.5%; PQI 16: -30.5%). The potential of improvement in the overall composite PQI (PQI 93) that can be achieved by each ACES can be found in Supplementary file 1 (Table S2).

**Table 5:** National Overall Potential of Improvement VRS DEA model by PQIs for Diabetes

**PQI**	**Actual PQI Rates (Average)**		**Optimal PQI Rates (Average)**	
	**2016**	**2017**	**2016**	**2017**
PQI 01: Diabetes short-term complications admission rate	19.5	14.6	7.3	5.1
PQI 03: Diabetes long-term complications admission rate	40.0	27.1	20.3	9.1
PQI 14: Uncontrolled diabetes admission rate	12.6	15.4	2.4	2.7
PQI 16: Lower-extremity amputation among patients with diabetes rate	13.1	11.2	5.9	4.1
PQI 93: Prevention quality diabetes composite	82.8	66.2	43.3	35.6

Abbreviations: VRS, vector return to scale; DEA, data envelopment analysis; PQI, prevention quality indicator.

## Discussion

 Primary care professionals’ activities include several different services, ranging from disease prevention, drug prescription, visits, and management of chronic diseases. In this study, we assessed primary care practices in managing diabetic patients. Several articles addressed the method for analyzing diabetes.^43,47^ We propose a novel and different approach of evaluating the quality indicators in PHC for diabetes in an integrated manner with the assessment of preventable hospitalizations. In particular, the study evaluates the way different practices deliver care to patients affected by this chronic disease.

 The production process considered in our study emphasizes the ideal use of human resources within ACES to produce better outcomes related to diabetes care, here summarized by avoidable hospitalization rates (PQIs). This efficiency assessment focusing on human resources is particularly relevant in the Portuguese health system, where gatekeeping is adopted and thus primary care providers, especially general practitioners, coordinate referrals to hospitals and specialists, and where a model of primary care unit called Family Health Units was introduced to promote group work as the main mode of provision of care, characterized by a higher cooperation between doctors, nurses and other PHC professionals.^63,64^

 Overall, assessing the DEA efficiency scores shows that improvement is still needed and possible for most of the ACES. In the first stage, it was found that most of the ACES were considered inefficient in different degrees. Individual ACES also presented great variability in efficiency according to the PQI used as output variable. The overall average efficiency scores worsened for PQI 01, 03 and 93 between 2016 and 2017. Furthermore, ACES performed worse for uncontrolled diabetes and lower-extremity amputation (PQIs 14 and 16), although these were the only indicators in which the overall mean efficiency improved in 2017.

 An important policy implication of this study could be that the technically inefficient ACES can, on average, improve their current diabetes related PQI rates by around 50% according to the VRS models. Differences regarding the effect of the scale assumption on the measurement of capacity utilization, however, have a theoretical background and should be considered. The CRS model reflects the fact that outputs will change by the same proportion as inputs are changed, whereas VRS models reflect production process or technologies that may exhibit increasing, constant and decreasing returns to scale. The efficiency frontier defines the full capacity output, in this case by means of PQI standardized rates, given a fixed level of inputs. The assumption of a CRS frontier is likely to result in a greater estimate of capacity output in comparison with VRS frontier.^65^ In the context of our study, however, VRS hypothesis seems to be more reliable, as it typically considers the effect on the relation between inputs and outputs and are more suitable when input or output variables are defined using ratios.^51^ In this sense, VRS frontier should better represent the production process involved in DM care at PHC level.

 According to inputs slack analysis, underutilization of human resources capacity is observed in most of the ACES. It is important in terms of performance to use the slack capacity pointed in the DEA models, particularly in the number of doctors and nurses, which registered considerably high slack values under the VRS assumption. It is important to notice that this study only used human resources, but there are other factors that may influence avoidable hospitalizations in diabetes. On a more macroscopic perspective, the results could be impacted by a variety of contextual factors intrinsic to each ACES, which represents distinct regions with particular characteristics, including socio-economic determinants, demographics, behavior patterns (smoking, diet, alcohol consumption, physical activity, obesity, etc)^66^ and burden of diseases. Nevertheless, in this study, we attempted to address this issue by including variables in the Tobit regression addressing regional characteristics, such as the proportion of elderly, burden of diseases (eg, prevalence of DM), socio-economic determinants such as unemployment rate, which is also proxy for income level, as well as several indicators regarding the management of diabetes at ACES level.

 The results showed, however, that only a few studied external factors may influence the efficiency of ACES with regard to avoidable hospitalizations related to DM, in which the proportion of patients with DM <65 years old with a HbA1c test ≤6.5% indicator was by far the most relevant factor, affecting efficiency in a positive way for most PQIs, and which corroborates with the results obtained in previous integrated analyses developed by the authors,^67^ apart from PQIs 1 and 14. Unemployment rate and prevalence of diabetes were the only determinants with a negative and statistically significant effect on efficiency, found for PQIs 1 and 16. The most surprising results, however, comes from the effects found for proportion of elderly, where an apparent positive and statistically significant effect on efficiency related to PQI 1 exists, which seems to contradict the conventional notion that increasing age would result in higher rates of avoidable hospitalization and thus would contribute to lower efficiency.

 In general, the estimated DEA efficiency scores can provide insights into performance in primary care practices, helping decision makers to detect problems and plan appropriate strategies to improve and adequate case by case based upon best practices (efficient providers). Primary care will continue to play a critical role in the next years to address current challenges and thus provide higher quality of life to the Portuguese population. This assessment is particularly timely as demographics and burden of chronic diseases are increasing the health needs. Furthermore, it provides evidence on potential of improvement and how the current set of primary care professionals’ activities can be used to reduce avoidable hospitalizations, promoting thereby a shift in resources from hospitals to primary care.^68,69^ Moreover, by using output-oriented models, it is possible to estimate the amount by which PQI rates could be improved (decreased) without enlarging the human resources. Some limitations, however, should be pointed in this study. A limitation of the DEA methodology relies on its deterministic nature, where the results are entirely dependent on the numeric values of inputs and outputs variables in the dataset. As the DEA model compares different DMUs to estimate relative efficiencies, the number and nature of DMUs present in the data can considerably change the results.^70^ For instance, if a more efficient DMU is added to the dataset, the frontier would be moved, and some of the efficiency scores of other DMUs would fall accordingly. Additionally, the use of a different set of input and output variables, such as other indicators related to diabetes as outputs or other input variables representing the professionals’ activities or resources (eg, number of PHC encounters) might have generated different conclusions. Another limitation is the comparability of ACES regarding certain dimensions of care, such as accessibility to primary care. Different ACES present distinct population density and size, resulting in important differences in the supply and demand of primary care services. An ACES serving much lower population density areas could be easily placed on the efficiency frontier for presenting a much lower number of patients and hospitalizations relatively to its peers. Another dimension that could be considered is continuity of care, which can be considered as the relationship between patients and primary care providers that goes beyond medical appointments and episodes of illnesses. This dimension can be included in the efficiency analysis by means of continuity indicators, such as size and mobility of patients between lists of general practitioners, distribution of the workload within the PHC professionals and communication with other healthcare sectors (referrals).^71^ Some primary data were not available at the time of conducting this study, such as the use of quality indicators applied to integrated management between the levels of care (primary, secondary, tertiary), quality of life indicators, environmental behaviour variables, and the composition of the medical team or other variables that reflect cooperation are essential factors. Therefore, the authors recommend an assessment as soon as possible. While it is essential to recognize that it is not possible to solve all these underlying issues, we attempted to minimize it by limiting the production process to primary care practices delivered by key primary care professionals and considering a second-stage analysis to understand the relevant factors contributing to efficiency. A sensitivity analysis considering variables that comprehensively reflect the complexity of the practices may be considered in the future, to overcome these limitations and to evaluate primary care efficiency in a broader scenario.

## Conclusion

 This study provided an empirical picture of the efficiency in managing diabetes at primary care level in 54 Portuguese health centre groupings (ACES). Results showed that inefficiency exists in the practices of most ACES, as well as the existence of a great variability among them with respect to efficiency scores. Inefficient ACES could substantially improve avoidable hospitalization rates due to diabetes using their current level human resources differently. Estimates showed that among the environmental determinants influencing the efficiency of the ACES in diabetes care, the proportion of patients with DM <65 years old with a HbA1c test result ≤6.5% correlates with efficiency in a positive way (that is, the higher the proportion of these patients, the lower the PQI rates are) for all PQIs, but PQIs 1 and 14. Our findings could be used for primary care managers and stakeholders to direct their attention to benchmarking their practices within their most efficient regional peers, or another comparative ACES, in order to understand their performance in a more detailed way. Furthermore, it narrows a gap in the literature in healthcare efficiency assessment, as there are very few disease-based DEA studies focusing on efficiency of healthcare. Our study suggests that efficiency assessment is a useful way to obtain insights into providers’ needs and potential of improvement in diabetes care at PHC level, and thus could be integrated into healthcare delivery performance assessments considering broader and integrated scopes.

## Acknowledgements

 The authors would like to thank the Central Authority for Health Services, for providing access to the PHC and hospital admission data. Also, the authors would like to thank the Project “1st.IndiQare - Quality indicators in primary health care: validation and implementation of quality indicators as an assessment and comparison tool,” financed by FEDER - European Fund - Regional Development Funds through the COMPETE 2020 - Operational Program for Competitiveness and Internationalization (POCI), and by Portuguese funds through FCT – Fundação para a Ciência e Tecnologia, for the support to carry out this study (POCI-01-0145-FEDER-030766).

## Ethical issues

 Data were provided by the Central Administration of the Health System, IP. (ACSS), an official organ of the Ministry of Health, through a cooperation protocol with the Faculty of Medicine, University of Porto and CINTESIS for research purposes.

## Competing interests

 Authors declare that they have no competing interests.

## Authors’ contributions

 Study conception by AR and AF. Study design and methodology by AR and JS. Material preparation and data collection by AR, and the analysis performed by AR and JS. Validation: PC, ML, PS, and AF. Writing – original draft by AR. Writing – review & editing: JS, PC, ML, PS, and AF. Supervision: PS and AF. Funding acquisition: Not applicable. All authors read and approved the final manuscript and are responsible for the correctness of the statements provided.

## Supplementary files

Supplementary file 1 contains Figures S1-S5 and Tables S1-S2.
