# Supplementary material for: Portuguese Primary Healthcare and Prevention Quality Indicators for Diabetes Mellitus – A Data Envelopment Analysis
Source: Int J Health Policy Manag. 2021 Jul 26;11(9):1725–34. doi: 10.34172/ijhpm.2021.76 (PMC9808229; doi:10.34172/ijhpm.2021.76)
Supplement: Supplementary file 1 — contains Figures S1-S5 and Tables S1-S2. [file ijhpm-11-1725-s001.pdf]

Article title: Portuguese Primary Health Care and Prevention Quality Indicators for Diabetes Mellitus – A Data Envelopment Analysis

Journal name: International Journal of Health Policy and Management (IJHPM)

Authors' information: Andre Ramalho[1,2]*, Julio Souza[1,2], Pedro Castro[3,2], Mariana Lobo[1,2], Paulo Santos[1,2], Alberto Freitas[1,2]

[1]MEDCIDS - Department of Community Medicine, Information and Health Decision Sciences, Faculty of Medicine, University of Porto, Porto, Portugal.

[2]CINTESIS - Centre for Health Technology and Services Research, Porto, Portugal.

[3]USF Camélias, ACES Gaia (Grande Porto VII - ARS Norte), Vila Nova de Gaia, Portugal.

(*Corresponding authors: andrelcramalho@gmail.com)

Supplementary file 1

Table S1. Estimation of the efficiency using Vector Return to Scale (VRS) DEA model with output orientation, according to the different models (supplement to Table 2)

ACES	PQI 01		PQI 03		PQI 14		PQI 16		PQI 93	
	2016	2017	2016	2017	2016	2017	2016	2017	2016	2017
Alentejo Central	3.58	4.37	3.59	7.11	6.66	3.83	2.23	3.81	2.57	2.59
Alentejo Litoral	1.00	1.00	1.00	1.00	1.00	1.00	1.00	1.00	1.00	1.00
Baixo Alentejo	2.09	4.90	2.39	5.17	3.96	1.52	3.21	3.18	2.75	2.09
São Mamede	4.54	2.44	1.37	3.88	9.84	1.69	2.18	1.79	2.40	1.42
Barlavento	3.42	4.67	2.45	4.58	1.01	3.88	3.51	1.94	1.98	2.07
Algarve Central	2.03	3.57	1.78	2.16	7.08	4.78	4.09	3.74	1.72	1.46

Sotavento	1.00	1.00	1.00	1.00	1.00	1.00	1.00	1.00	1.00	1.00
Baixo Mondego	1.79	1.61	1.78	2.30	30.65	7.54	2.71	2.96	2.17	1.22
Baixo Vouga	2.10	3.49	1.76	2.30	9.93	7.82	3.31	2.53	1.71	1.55
Beira Interior Sul	1.00	1.00	1.00	1.37	1.00	1.58	1.00	1.00	1.00	1.00
Cova da Beira	1.00	2.21	1.00	2.58	1.00	10.84	1.00	2.67	1.00	2.70
Dão Lafões	2.12	3.33	1.67	3.09	12.05	9.84	2.10	2.16	1.53	1.64
Guarda	1.00	1.00	1.00	3.07	4.49	6.95	1.76	1.83	1.01	1.46
Pinhal Interior Norte	1.00	1.59	1.68	1.95	19.99	3.06	1.30	1.00	2.10	1.00
Pinhal Litoral	2.60	3.41	2.93	2.71	43.50	15.58	2.06	2.59	2.84	2.25
Almada/ Seixal	3.03	4.59	2.42	3.57	5.24	6.47	3.66	4.60	2.04	1.88
Amadora	7.09	5.21	3.05	4.14	7.10	5.68	3.24	6.64	3.01	2.73
Arco Ribeirinho	3.94	2.28	3.39	4.57	11.77	15.14	2.56	6.92	2.61	2.81
Arrábida	3.59	4.33	3.02	8.11	5.94	5.87	3.81	7.79	2.25	2.83
Cascais	3.48	3.47	2.51	5.64	8.85	5.29	1.53	5.28	1.97	2.35
Estuário do Tejo	4.26	5.72	2.88	4.91	22.20	21.31	3.23	8.69	2.73	3.73
Lezíria	5.00	7.02	2.39	2.37	23.07	14.49	3.22	4.80	2.77	2.89
Lisboa Central	2.54	2.80	2.82	4.57	23.38	11.45	1.50	2.33	2.48	2.38
Lisboa Norte	3.44	3.46	2.14	3.53	18.79	12.84	2.10	3.24	2.21	2.31
Lisboa Ocidental e Oeiras	3.78	3.98	2.78	3.39	14.72	6.70	2.51	3.82	2.34	1.98
Loures/Odivelas	3.65	2.66	2.06	2.33	13.21	6.65	3.03	2.31	2.12	1.41
Médio Tejo	2.84	3.57	1.16	3.20	16.50	7.52	1.66	3.39	1.59	1.90
Oeste Norte	4.35	4.53	2.55	4.55	21.39	10.93	3.12	5.19	2.54	2.87
Oeste Sul	3.03	1.69	2.91	4.80	6.18	6.16	3.81	3.30	2.22	1.89
Sintra	5.28	5.48	3.38	3.87	14.06	7.24	4.76	7.37	3.13	2.41
Alto Ave	4.25	5.63	1.84	2.32	6.94	12.03	2.45	4.62	1.90	2.13
Alto Minho	1.87	2.98	2.08	3.96	5.01	4.92	1.70	4.38	1.48	1.77
Alto Tâmega e Barroso	7.94	3.87	2.75	3.86	7.40	9.38	1.91	1.75	3.07	2.22
Ave/Famalicão	1.00	1.77	1.00	1.79	1.00	1.60	1.00	1.50	1.00	1.18
Aveiro Norte	1.78	1.65	1.00	3.36	3.23	2.46	1.99	1.54	1.16	1.27

Baixo Tâmega	2.05	1.61	1.41	4.19	1.67	1.57	1.10	1.38	1.35	1.40
Barcelos/Esposende	2.62	1.62	1.63	1.79	8.72	4.74	3.07	3.48	1.84	1.31
Braga	2.83	2.90	1.36	1.36	7.22	1.00	1.83	3.24	1.36	1.05
Douro Sul	2.45	1.71	1.49	2.04	8.37	9.08	1.57	1.00	2.02	1.84
Espinho Gaia	4.25	5.09	1.07	2.03	11.52	3.05	1.00	2.07	1.51	1.51
Feira e Arouca	2.62	2.31	1.36	2.56	5.92	3.67	1.74	2.85	1.48	1.37
Gaia	2.77	4.05	1.12	2.56	8.77	3.97	1.89	3.95	1.40	1.72
Gerês/Cabreira	3.75	2.68	2.11	1.00	6.77	1.56	3.41	1.31	2.31	1.00
Gondomar	3.62	4.26	3.76	6.50	11.54	7.05	2.70	5.76	2.78	2.76
Maia/Valongo	2.40	3.26	3.47	7.78	23.54	13.55	2.99	4.83	2.71	3.18
Marão e Douro Norte	2.53	1.18	1.89	3.79	13.11	8.10	1.15	1.69	1.91	1.71
Matosinhos	2.76	3.11	2.58	5.61	6.70	6.74	3.35	5.80	2.05	2.39
Nordeste	2.69	3.70	1.71	2.16	10.43	3.92	1.57	3.26	1.54	1.45
Porto Ocidental	1.66	3.47	3.26	5.18	15.93	6.36	2.63	5.57	2.41	2.32
Porto Oriental	1.53	2.45	3.05	4.36	12.40	7.93	3.09	1.71	2.33	2.24
Vila do Conde/Póvoa de Varzim	2.69	2.02	2.83	4.56	16.14	9.80	2.47	2.98	2.33	2.03
Santo Tirso/Trofa	1.37	1.00	1.23	1.00	2.66	1.00	2.09	1.00	1.23	1.00
Vale do Sousa Norte	2.59	3.82	2.51	4.75	1.00	5.88	2.03	2.14	1.76	2.23
Vale do Sousa Sul	3.61	2.29	1.62	5.45	4.08	4.93	1.78	1.11	1.57	1.83

Table S2. Potential of improvement Vector Return to Scale (VRS) DEA model. Target: PQI 93 rates (supplement to Table 5)

ACES	Actual PQI 93 2016	VRS DEA 2016	Actual PQI 93 2017	VRS DEA 2017
Alentejo Central	99.52	38.76	83.64	32.32
Alentejo Litoral	75.42	75.42	87.35	87.35
Baixo Alentejo	198.52	72.14	89.95	43.12
São Mamede	100.80	42.00	48.20	33.86
Barlavento	76.89	38.76	69.39	33.60

Algarve Central	66.57	38.76	47.23	32.32
Sotavento (Efficient)	38.76	38.76	41.82	41.82
Baixo Mondego	84.09	38.76	39.37	32.32
Baixo Vouga	66.18	38.76	50.06	32.32
Beira Interior Sul	63.52	63.52	35.48	35.48
Cova da Beira	123.06	123.06	100.27	37.12
Dão Lafões	59.11	38.76	52.97	32.32
Guarda	63.70	63.09	63.11	43.12
Pinhal Interior Norte	81.23	38.76	32.32	32.32
Pinhal Litoral	109.99	38.76	72.85	32.32
Almada/ Seixal	79.04	38.76	60.79	32.32
Amadora	116.68	38.76	91.03	33.38
Arco Ribeirinho	101.13	38.76	90.66	32.32
Arrábida	87.36	38.76	91.60	32.32
Cascais	76.31	38.76	75.86	32.32
Estuário do Tejo	106.01	38.76	120.58	32.32
Lezíria	107.36	38.76	93.35	32.32
Lisboa Central	96.10	38.76	76.82	32.32
Lisboa Norte	85.64	38.76	74.81	32.32
Lisboa Ocidental e Oeiras	90.57	38.76	63.86	32.32
Loures/Odivelas	82.03	38.76	45.63	32.32
Médio Tejo	61.48	38.76	61.31	32.32
Oeste Norte	98.26	38.76	92.67	32.32
Oeste Sul	86.03	38.76	61.12	32.32
Sintra	121.26	38.76	77.99	32.32
Alto Ave	73.75	38.76	68.68	32.32
Alto Minho	57.19	38.76	57.22	32.32
Alto Tâmega e Barroso	118.88	38.76	76.16	34.31
Ave/Famalicão	72.14	72.14	68.66	58.23

Aveiro Norte	45.10	38.76	44.04	34.58
Baixo Tâmega	52.48	38.76	45.86	32.82
Barcelos/Esposende	71.14	38.76	43.47	33.07
Braga	52.85	38.76	33.84	32.32
Douro Sul	78.25	38.76	67.36	36.67
Espinho Gaia	58.55	38.76	48.89	32.32
Feira e Arouca	57.41	38.76	44.62	32.60
Gaia	54.33	38.76	56.78	32.99
Gerês/Cabreira	89.59	38.76	34.41	34.41
Gondomar	107.89	38.76	89.22	32.32
Maia/Valongo	105.10	38.76	102.85	32.32
Marão e Douro Norte	74.23	38.76	57.49	33.66
Matosinhos	79.60	38.76	77.38	32.32
Nordeste	59.80	38.76	46.94	32.32
Porto Ocidental	93.29	38.76	75.04	32.32
Porto Oriental	90.31	38.76	80.08	35.68
Vila do Conde/Póvoa de Varzim	90.38	38.76	67.10	32.99
Santo Tirso/Trofa	56.43	45.83	67.73	67.73
Vale do Sousa Norte	68.21	38.76	72.06	32.32
Vale do Sousa Sul	60.99	38.76	59.25	32.32

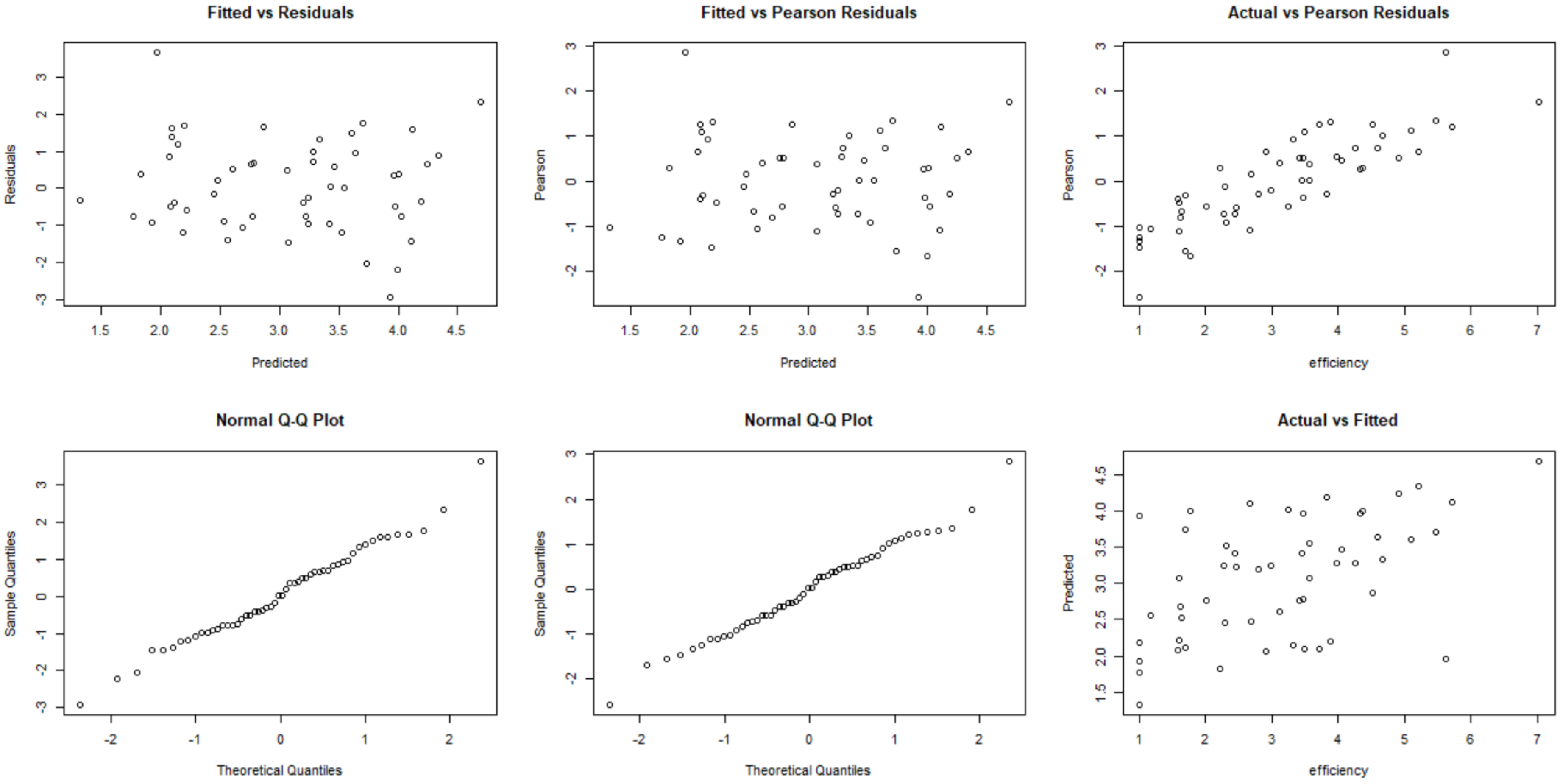

Figure S1. Basic residual plots for checking the Tobit Regression model fit - PQI 01.

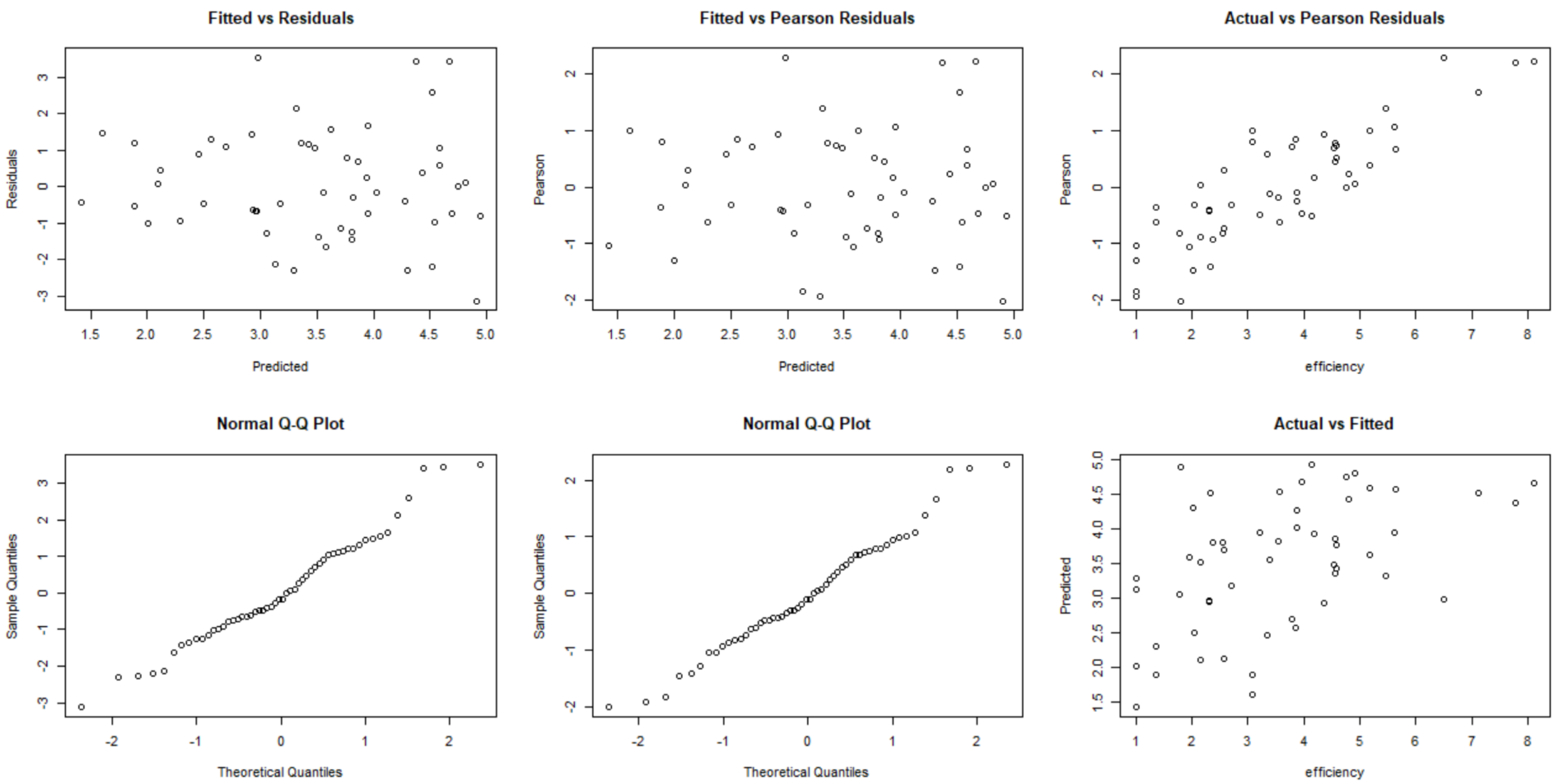

Figure S2. Basic residual plots for checking the Tobit Regression model fit – PQI 03.

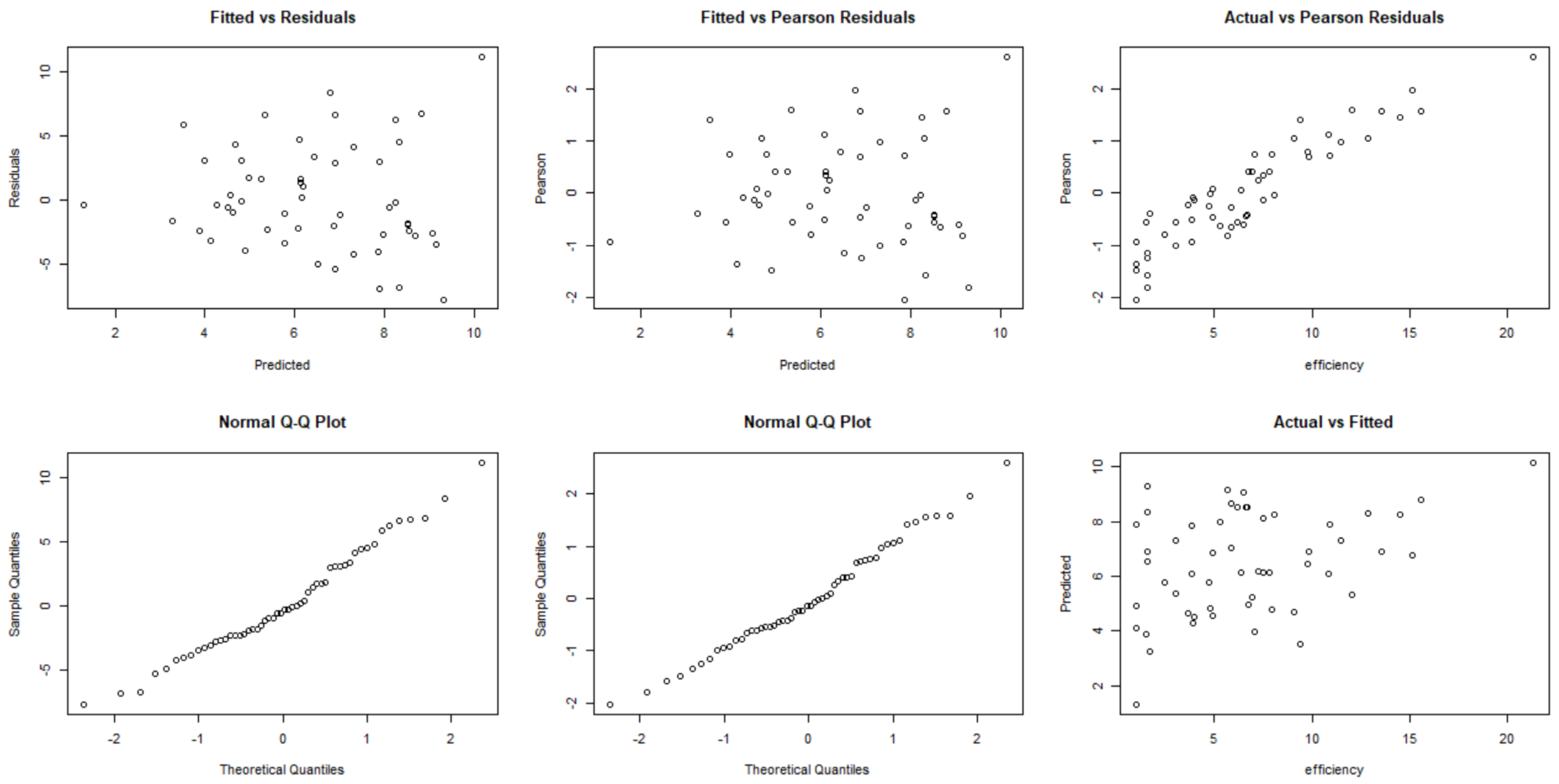

Figure S3. Basic residual plots for checking the Tobit Regression model fit – PQI 14.

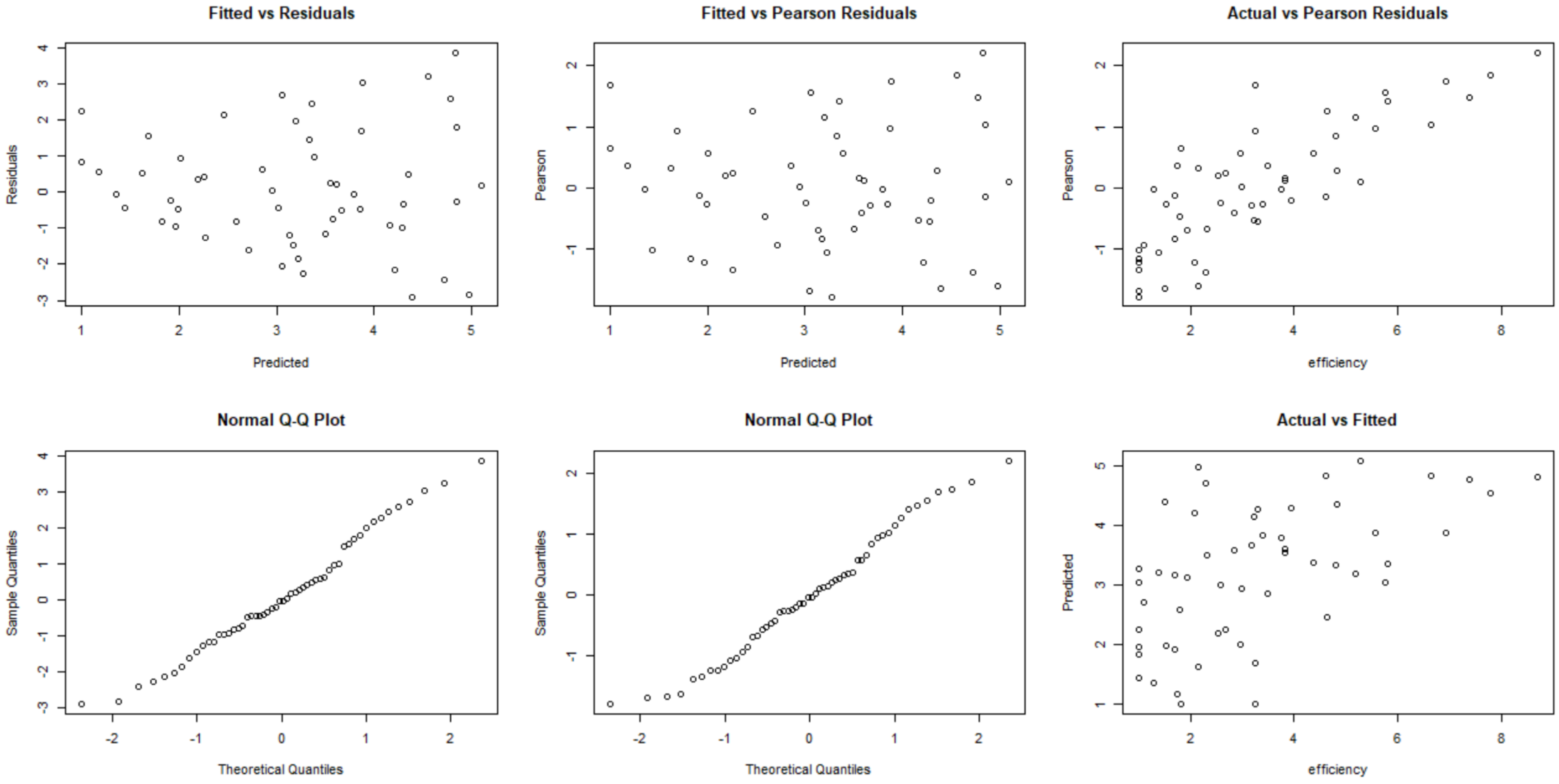

Figure S4. Basic residual plots for checking the Tobit Regression model fit – PQI 16.

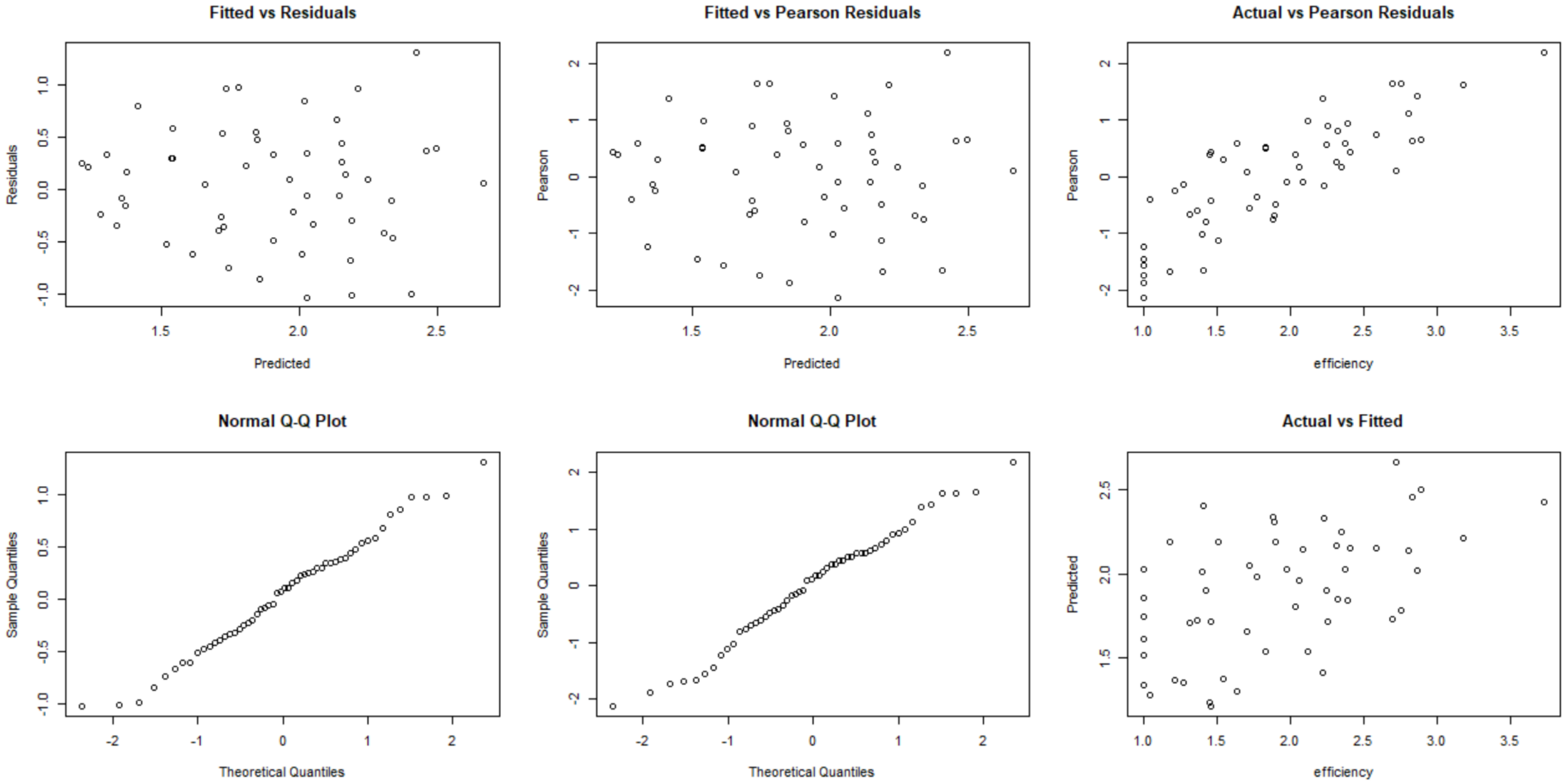

Figure S5. Basic residual plots for checking the Tobit Regression model fit – PQI 93**.**
